# Developing Screen-Printing Processes for Silver Electrodes Towards All-Solution Coating Processes for Solar Cells

**DOI:** 10.3390/polym16213012

**Published:** 2024-10-27

**Authors:** Tsui-Yun Chung, Hou-Chin Cha, Chih-Min Chuang, Cheng-Si Tsao, Damian Glowienka, Yi-Han Wang, Hui-Chun Wu, Yu-Ching Huang

**Affiliations:** 1 Department of Materials Engineering, Ming Chi University of Technology, New Taipei City 24301, Taiwan; tychung@mail.mcut.edu.tw (T.-Y.C.); wuhc@mail.mcut.edu.tw (H.-C.W.); 2 Organic Electronics Research Center, Ming Chi University of Technology, New Taipei City 24301, Taiwan; ennowang@mail.mcut.edu.tw; 3 College of Engineering, Ming Chi University of Technology, New Taipei City 24301, Taiwan; 4 Department of Physics, National Atomic Research Institute, Taoyuan 32546, Taiwan; cmchuang@nari.org.tw; 5 Department of Materials Science and Engineering, National Taiwan University, Taipei City 10617, Taiwan; tsaochengsi@gmail.com; 6 Faculty of Applied Physics and Mathematics, Gdańsk University of Technology, Narutowicza 11/12, 80-233 Gdańsk, Poland; damian.glowienka@pg.edu.pl; 7 Department of Chemical and Materials Engineering, College of Engineering, Chang Gung University, Taoyuan 33302, Taiwan

**Keywords:** screen printing, organic photovoltaic, perovskite solar cell, near-infrared annealing, slot-die coating

## Abstract

In recent years, third-generation solar cells have experienced a remarkable growth in efficiency, making them a highly promising alternative energy solution. Currently, high-efficiency solar cells often use top electrodes fabricated by thermal evaporation, which rely on high-cost and high energy-consumption vacuum equipment, raising significant concerns for mass production. This study develops a method for fabricating silver electrodes using the screen-printing process, aiming to achieve solar cell production through an all-solution coating process. By selecting appropriate blocking-layer materials and optimizing the process, we have achieved device efficiencies for organic photovoltaics (OPVs) with screen-printed silver electrodes comparable to those with silver electrodes fabricated by thermal evaporation. Furthermore, we developed a method to cure the silver ink using near-infrared (NIR) annealing, significantly reducing the curing time from 30 min with hot air annealing to just 5 s. Additionally, by employing sheet-to-sheet (S2S) slot-die coating, we scaled up the device area and completed module development, successfully verifying stability in ambient air. We have also extended the application of screen-printed silver electrodes to perovskite solar cells (PSCs).

## 1. Introduction

As the global warming crisis intensifies, energy sources with low carbon emissions are gaining increasing attention. Consequently, renewable solar energy is highly anticipated as a key solution. The third generation of solar cell technology has seen rapid advancements in recent years, with organic photovoltaics (OPVs) and perovskite solar cells (PSCs) experiencing the most remarkable growth. OPVs have become a widely researched topic due to their low manufacturing cost, low carbon footprint, flexibility, and solution processability [1,2,3,4]. Solution-processed OPVs based on blends of donor and acceptor materials with a bulk heterojunction (BHJ) structure exhibit excellent charge separation and transport behaviors. With the rapid progress in the synthesis of low-bandgap donor materials, the highest power conversion efficiency (PCE) has now surpassed 19% [5,6]. On the other hand, PSCs have garnered significant attention, due to their outstanding PCE. The high PCE of PSCs is attributed to their strong light absorption [7], low exciton-binding energy, and long carrier diffusion length [8]. The certified PCE of PSCs has rapidly increased from 3.8% in 2009 to 26.2% [9] within 15 years. The bandgap of PSCs can be tuned to combine with inorganic solar cells to create silicon/PSC tandem solar cells, achieving high PCEs of over 30%. These high-PCE tandem solar cells are considered an emerging technology that will significantly impact the future solar industry [10,11]. However, the high-efficiency devices mentioned above were all achieved in laboratory conditions with extremely small active areas, and most of them utilized high-vacuum thermal evaporation processes to fabricate the metal top electrodes. This approach contradicts the current international goal of reducing carbon emissions. Therefore, developing solution processes has become an essential solution.

Therefore, to promote the commercialization of OPVs and PSCs, various wet deposition technologies suitable for large-scale production already exist, including spray coating [12,13], inkjet printing [14,15], slot-die coating [16,17,18,19], gravure printing [20,21], and screen printing [22,23]. However, most research focuses on depositing the hole-transport layer (HTL), electron transport layer (ETL), and active layer, rather than the top metal electrode. Top metal electrodes such as aluminum (Al), silver (Ag), and gold (Au) are typically fabricated via thermal evaporation in a high vacuum chamber. Vacuum-based deposition techniques are widely used for large-scale production, due to their ability to accommodate a broad range of materials. While effective, vacuum deposition of metals is known for its high costs. In contrast, completing metal electrode fabrication through solution-based processes, such as screen printing, offers greater potential for integration into continuous methods like roll-to-roll manufacturing. This approach can significantly reduce both fabrication time and production costs, making it highly advantageous for scaling up solar cell production. While the material options for the screen-printing process are quite limited, making it less ideal for optimizing the contact interfaces in high-efficiency solar cells, it is crucial to consider the interface between the active layer and the metal electrode during the fabrication of metal electrodes via screen printing. To ensure efficient charge transport and minimize the potential drawbacks of the screen-printing process, an additional interfacial layer is often required to optimize the contact and improve overall device performance.

However, printing processes typically require post-deposition treatments such as drying, curing, or annealing, which are conventionally performed through heating, thus extending production timelines. Therefore, developing faster post-deposition processing methods for metal inks is crucial to reducing production time and improving overall efficiency in mass production. Moreover, screen-printed inks typically require curing at high temperatures for several tens of minutes. This prolonged high-temperature treatment would damage the active layer and reduce the performance of solar cells. Therefore, developing an appropriate processing method to overcome these challenges is essential. Near-infrared (NIR) is a suitable heat treatment method for rapid processing [24,25,26,27,28,29,30]. Using NIR heating can rapidly raise the temperature, significantly reducing the curing time of the silver electrode while avoiding damage to the underlying active layer and other cell structures. Compared to traditional heating methods, NIR heating is more energy-efficient, thus lowering production costs. Additionally, NIR heating allows for more uniform curing of the silver electrode, thereby improving conductivity and the overall performance of the device. This method can also be applied to different production lines and scales, making it suitable for large-scale manufacturing.

The main objective of this study is to achieve the fabrication of solar cells using an all-solution process by depositing the silver electrode through screen printing. We first focused on OPVs to evaluate screen-printed silver electrodes, systematically analyzing the feasibility of using blocking-layer materials for these electrodes. Previous studies have used inkjet printing [31] with a 5 min sintering time or screen printing [32] with a 30 min sintering time. In contrast, our approach combines NIR heating with screen printing to achieve rapid sintering, significantly improving processing efficiency. We carefully verified the PCE differences between the devices fabricated from screen-printed silver electrodes and thermally-evaporated silver electrodes. To reduce processing time, we have developed suitable NIR processing parameters for curing screen-printed electrodes to ensure that the efficiency of the devices is comparable to that of the reference devices. Using a sheet-to-sheet (S2S) slot-die coating process to fabricate the electron transport layer, active layer, and hole-transport layer, combined with screen-printed silver electrodes, we demonstrated fully solution-processed solar cells. For commercialization purposes, we further scaled up the device area and fabricated OPV modules. Additionally, we examined the durability of the devices with electrodes prepared using the screen-printing method. Finally, we applied this process to PSCs to demonstrate the versatility of the screen-printing method for fabricating silver electrodes.

## 2. Experiments

### 2.1. Materials

Indium tin oxide (ITO)-coated glass substrate as the transparent electrode was purchased from Optical Filter Ltd. (Thame, UK) (EMI-ito 15, surface resistance of 15 Ω). The ITO glass was cleaned with acetone (Acros, Geel, Belgium, Mw: 58.08 g/mol, 99%) and IPA (Acros, Mw: 60.1 g/mol, 99%) sequentially, in an ultrasonic tank. Zinc acetate and aluminum acetate were obtained from Alfa Aesar and Aldrich, respectively. The surfactant Zonyl FS-300 was purchased from Fluka (Charlotte, NC, USA). Polyethylenimine ethoxylated (PEIE) received from Aldrich was diluted in 2-methoxyethanol into 0.4 wt% of solution. The precursor of aluminum-doped zinc oxide (AZO) solution was prepared by dissolving zinc acetate, aluminum acetate, and Zonyl FS-300 in deionized water (DIW). For the sheet-to-sheet (S2S) slot-die coating process, the as-prepared AZO precursor was first filtered through a 0.45 μm filter, then diluted with DIW at a 1:1 volume ratio. After that, the AZO precursor was mixed with 20 vol% of PEIE to form a hybrid AZO:PEIE_20_ solution. P3HT (Mw: 30–40 k g/mol, PDI: ~2.0), and PCBM (Mw: 910 g/mol, 99.5%) was obtained from Rieke Metals (Lincoln, NE, USA). The P3HT:PCBM solution was prepared by dissolving 15 mg of P3HT and 15 mg of PCBM in 1 mL of o-xylene (Alfa Aesar, Mw: 106.2 g/mol, 99%) and stirred at 45 °C overnight for preparing the photoactive layer solution. PEDOT:PSS solution (Clevios CPP 105D) used for OPVs was purchased from Sigma-Aldrich (St. Louis, MO, USA). Nickel acetate tetrahydrate (NiAc_,_ 99%) was purchased from SHOWA Chemical (Tokyo, Japan). Lead iodide (PbI_2_, 99.9985%), and Methylammonium iodide (MAI, 99.9985%) were purchased from Alfa Aesar (Ward Hill, MA, USA). For the hole-transport layer, 124.40 mg nickel acetate tetrahydrate was dissolved in 1 mL of ethanol, and then the solution was stirred at 60 °C until it became clear. After adding 30 μL of ethanolamine, the solution was filtered with 0.45 μm filter. Polyethylenimine (PEI), dimethylformamide (DMF, 99.8%), dimethyl sulfoxide (DMSO, >99.9%), Chlorobenzene (CB, >99.0%), and ether were purchased from Sigma-Aldrich. The PCBM solution was prepared by dissolving 20 mg of PCBM in 1 mL of CB and stirred at 70 °C overnight for preparing the electron transport-layer solution. Diethyl ether (99.0%) and ethyl alcohol (EtOH 99.99%) were obtained from Fisher. PEDOT:PSS solution (Clevios F010) used for PSCs was purchased from Sigma-Aldrich.

### 2.2. Device Fabrication

We slot-die coated the AZO:PEIE_20_ precursor as the ETL and P3HT:PCBM as the photoactive layer by using the Coatema S2S system (Coatema easycoater, Dormagen, Germany). Before the ETL deposition, the ITO-coated glass substrate was ultrasonically cleaned in a series of organic solvents (methanol, acetone, and isopropanol), and then treated with O_2_ plasma for 3 min. For the ETL deposition, the coating speed, solution flux, and substrate temperature were set at 0.5 m/min, 0.2 mL/min, and 40 °C, respectively. After depositing the AZO precursor, the ITO/AZO substrates were heated at 150 °C for 10 min in an ambient oven. Next, the photoactive layer of P3HT:PCBM was slot-die coated on the ITO/AZO substrate with a coating speed of 2 m/min, solution flux of 1.5 mL/min, and a substrate temperature of 60 °C. The PEDOT:PSS was spin-coated as HTL on the P3HT:PCBM film. For the reference devices, we thermally evaporated the silver electrode. In addition, we screen-printed the silver ink on the PEDOT:PSS layer and tuned the NIR curing parameters, including the irradiation distance, curing power, and time. The device area is defined by the area of the metal electrode. It is noteworthy to mention that all the S2S coating processes were conducted in air, and none of the devices were encapsulated.

The NiO_X_ solution was spin-coated onto the clean FTO glasses at 4000 rpm for 20 s, followed by 20 min annealing in the air at 300 °C. The perovskite precursor solution was prepared by dissolving MAI and PbI_2_ in a molar ratio of 1:1 in 1 mL of a solvent mixture of DMF/DMSO (5:2 by volume). The perovskite films were spin-coated from the precursor onto the prepared ITO/NiOx substrate in a glovebox at 4500 rpm for 30 s. At the 15th second, 300 μL of diethyl ether was dropped on the spin-coated perovskite film. The perovskite films were annealed on the hot plate for 2 min at 100 °C to form a dark brown perovskite film. After that, PCBM was spin-coated at 1000 rpm for 20 s onto the perovskite film to form the electron transport layer. The SnO_2_ NPs layer was then spin-coated at 3000 rpm for 30 s, followed by thermal treatment on a hot plate at 70 °C for 10 min. After initial screening, we selected PEDOT:PSS (Clevios F010) as the blocking-layer material. PEDOT:PSS was applied using a spin-coating process at 5000 rpm for 5 s, then baked at 100 °C in air for 3 min, followed by an additional 10 min of baking in a glovebox to remove residual water. The top electrode was applied using either screen printing in air or thermal evaporation in a vacuum chamber. The silver paste used for the top electrode, fabricated using the screen-printing process, is Dupont PV416 (Durham, NC, USA), a commercially available silver paste. The paste has a solid content of 81–84% and a viscosity ranging from 90 to 130 Pa·s. A 280-mesh stainless steel composite screen was selected for the printing process. After depositing the PV416 via screen printing, the paste was baked in a hot air oven at 80 °C for 30 min.

### 2.3. Characterization

Current density–voltage curves were measured by using a solar simulator (Abet technologies, Model #11000) under A.M. 1.5 illumination (100 mW/cm^2^) in the ambient air. Film thicknesses were measured using a profilometer (Alpha Step D-100, KLA Tencor). The topography of the screen-printed silver was measured by scanning electron microscopy (SEM, Hitachi S-4800). The NIR equipment was purchased from MOS Technology Inc. (NIR-252, Zhubei City, Taiwan). It is equipped with six lamps, each with an irradiation power of 2.1 kW. The wavelength range of the light source is 500–1500 nm, with a peak emission between 700 and 900 nm. The irradiation power can be adjusted between 10% and 100%. The illumination area is 25 × 25 cm^2^, and the irradiation height can be adjusted within a range of 2–20 cm. The sheet resistance was measured using a custom-developed system, utilizing a four-point probe method for accurate measurement.

## 3. Results and Discussion

**Figure 1:**
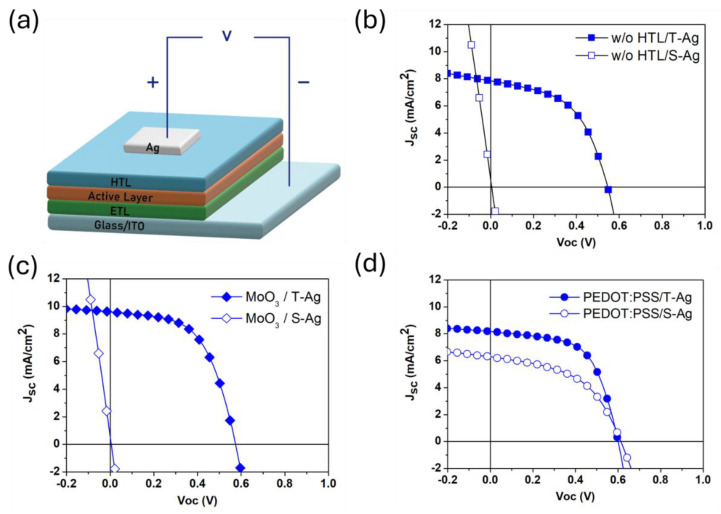
(**a**) The schematic of the device architecture, and the J–V curves of TAg and S-Ag devices: (**b**) without a blocking layer (HTL), and with (**c**) MoO_3_ and (**d**) PEDOT:PSS as the blocking layer (HTL).

Firstly, to clarify the effect of the solvent in silver paste on the underlying active layer, we fabricated inverted OPV devices with the following structure: ITO/AZO:PEIE_20_/P3HT:PCBM/HTL/Ag. The device architecture is shown in Figure 1a, where different types of hole-transport layers were selected to compare their effects on cell performance. The silver electrodes were prepared using thermal evaporation and screen-printing processes, respectively, to compare the effects of different fabrication methods on the efficiency of the devices. The current density–voltage (J–V) curves are shown in Figure 1b. The devices with thermally evaporated silver electrodes are referred to as T-Ag, while those with screen-printed silver electrodes are referred to as S-Ag. Typically, when fabricating silver electrodes via thermal evaporation without an HTL between the active layer and the metal electrode, the device needs to be exposed to the atmosphere for a period, to form a thin layer of metal oxide on the surface. This oxide layer improves the alignment between the electrode and the energy level of the active layer, allowing for a normal PCE. As a result, the PCE of the T-Ag device without HTL in Figure 1b was 2.2%, while the efficiency of the S-Ag device without HTL could not be measured, due to a short circuit, as reflected in the J-V curve. MoO_3_ is a commonly used HTL material in inverted OPV structures. We first fabricated the MoO_3_ HTL via thermal evaporation and then compared the PCE differences between the T-Ag and S-Ag devices. The J-V curves are shown in Figure 1c. The average efficiency of T-Ag devices with an 8 nm MoO_3_ HTL was 3.10%, representing approximately a 40% increase compared to T-Ag devices without an HTL. This confirms that the presence of MoO_3_ allows the device to achieve optimal performance immediately after fabrication. Next, we fabricated S-Ag devices by screen printing on top of the 8 nm MoO_3_ layer. However, these devices still exhibited short-circuit behavior. Due to the nature of MoO_3_ as a metal oxide, increasing its thickness further would significantly reduce its conductivity, negatively impacting overall performance. Thus, while metal oxide HTLs offer good hole-transport properties at certain thicknesses, they are not suitable for the screen-printing process, as they cannot effectively prevent the detrimental effects of the silver paste on device performance.

Using conductive polymers as the HTL is another option for preparing OPV devices. In this study, PEDOT:PSS (Clevios CPP 105D) was chosen as the HTL. Firstly, a layer of PEDOT:PSS film with a thickness of approximately 40–60 nm was spin-coated onto the active layer. Subsequently, silver electrodes were prepared using thermal evaporation and screen-printing processes, respectively, to evaluate the suitability of PEDOT:PSS as the HTL for screen-printed Ag electrodes. The J-V curves and parameters for the device with PEDOT:PSS as the HTL are shown in Figure 1d and Table 1. For T-Ag devices with PEDOT:PSS as the HTL, the initial average efficiency was 2.70%, which is a 23% increase compared to T-Ag devices without an HTL. Notably, for S-Ag devices with PEDOT:PSS as the HTL, the average efficiency reached 1.77%, showing some effectiveness of PEDOT:PSS as an HTL in blocking the penetration of silver paste. However, the efficiency of S-Ag devices is still approximately 35% lower than that of T-Ag devices, which is attributed to inappropriate PEDOT:PSS film thickness and silver paste-curing parameters. From the results, it is evident that using PEDOT:PSS as the HTL in S-Ag devices can successfully block the penetration of silver paste, preventing damage to the underlying active layer. Therefore, optimizing the thickness of the PEDOT:PSS layer to achieve the best electrical and blocking properties should be a key factor in improving the efficiency of S-Ag devices. To understand the buffering effect of the HTL layer thickness on screen-printed silver paste, subsequent tests will be conducted using slot-die coating to produce PEDOT:PSS layers of varying thicknesses for verification.

**Figure 2:**
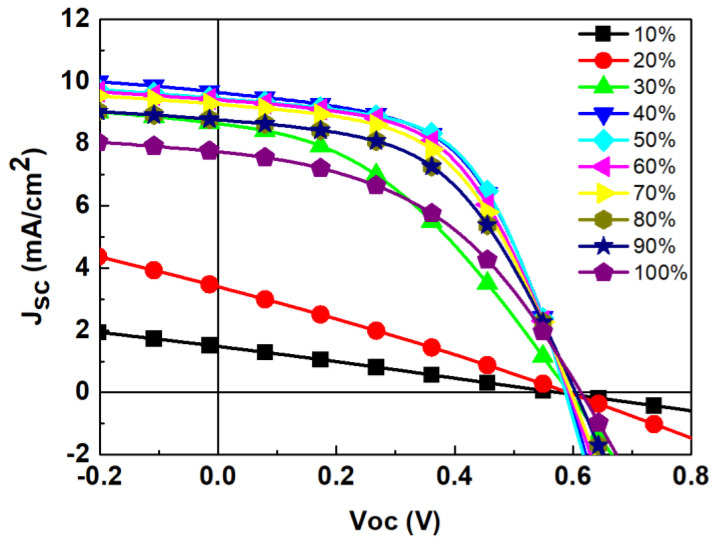
J–V curves of S-Ag devices under different NIR irradiation intensities.

In the preparation of S-Ag devices using screen printing, the silver paste requires curing, which also involves thermal treatment of the active layer in the device. Inappropriate thermal treatment parameters can damage the structure of the active layer, leading to a decrease in device efficiency. Therefore, the curing parameters of the silver paste are crucial factors affecting device efficiency. When using conventional heating methods for silver paste curing, we adopted curing conditions of heating at 80 °C for 30 min. However, this method requires a long duration to achieve optimal electrical properties. Thus, developing process conditions using NIR heating can achieve the goal of rapidly producing OPVs. The NIR irradiation parameters were designed by exposing the sample at a fixed height (the distance between the light source and the sample) to test the effects of different irradiation energies (as a percentage of the maximum NIR light-source intensity) on the electrical properties of the silver electrodes and device efficiency. In this study, the irradiation height was set at 15 cm, the exposure time was 5 s, and the irradiation intensity was tested in a range from 10% to 100%. The results are shown in Figure 2 and Table 2. As the NIR irradiation energy increased from 10% to 30%, the PCE of the S-Ag devices gradually improved. When the irradiation intensity reached 40%, there was a significant increase in the PCE of the S-Ag devices. Observations revealed that at irradiation energies below 40%, the silver paste exhibited areas that were not fully dried, indicating incomplete curing, which led to lower device performance. At an irradiation energy of 40%, the PCE of the S-Ag devices significantly increased, to 2.90 ± 0.20%. The optimal NIR irradiation energy range for fabricating S-Ag devices was found to be between 40% and 60%, where the efficiency of the S-Ag devices was comparable to that of the T-Ag devices (3.08 ± 0.05)%. This indicates that this irradiation energy range allows for complete curing of the silver paste without degrading the other layers in the cell. However, when the irradiation energy exceeded 70%, the PCE of the S-Ag devices sharply decreased, likely due to excessive NIR irradiation energy.

**Figure 3:**
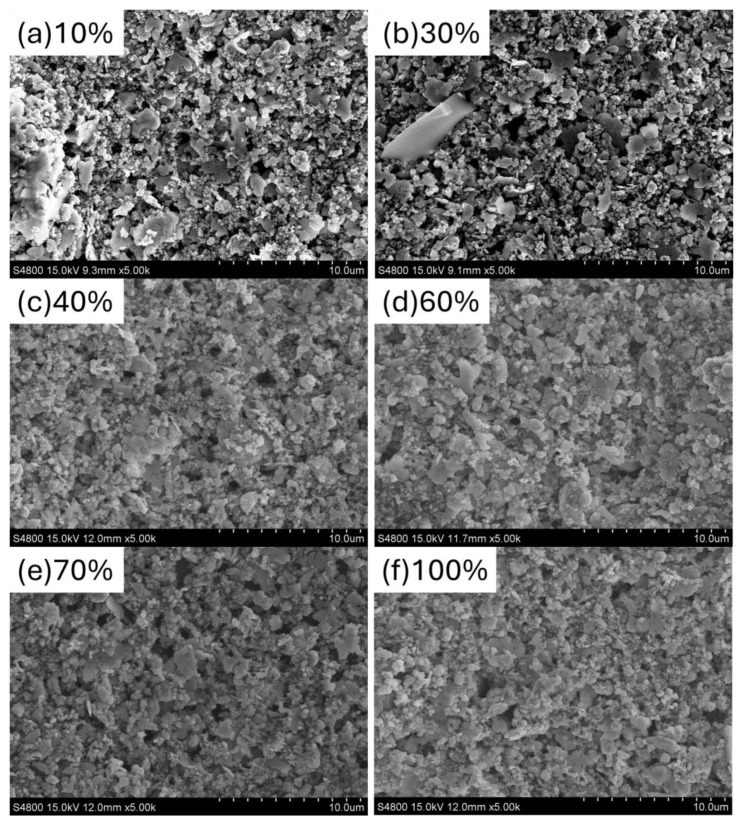
SEM images of the OPV top electrodes under different NIR irradiation intensities: (**a**) 10%, (**b**) 30%, (**c**) 40%, (**d**) 60%, (**e**) 70%, (**f**) 100%.

We further measured the surface morphology of the screen-printed silver electrodes after NIR irradiation. Figure 3 shows the surface morphology of the top electrodes after exposure to different NIR irradiation intensities. Additionally, to clarify the situation with only silver electrodes, the data for the sheet resistance value as it varies with NIR heating have also been included, as shown in Table 3. When the NIR irradiation intensity was 10% and 30%, the surface morphology did not show large-scale silver particle aggregation and exhibited a porous structure, indicating incomplete curing of the silver paste at these irradiation intensities. When the NIR irradiation intensity increased to 40%, the pores in the surface morphology significantly decreased, and the silver particles showed larger-scale agglomeration, forming a sheet-like film. Four-point probe measurements revealed that the sheet resistance of electrodes cured at 40% NIR irradiation intensity (0.3 Ω) was almost equivalent to that of silver electrodes prepared by thermal evaporation (0.39 Ω, ref). As the NIR irradiation intensity increased from 70% to 100%, the surface morphology of the electrodes showed larger and more uniform silver-particle agglomeration. Although the surface morphology of the silver electrodes did not change significantly at these higher irradiation intensities, the PCE of the S-Ag devices began to drop sharply. This decline is likely due to the excessive NIR irradiation intensity damaging the other layers of the OPVs.

**Figure 4:**
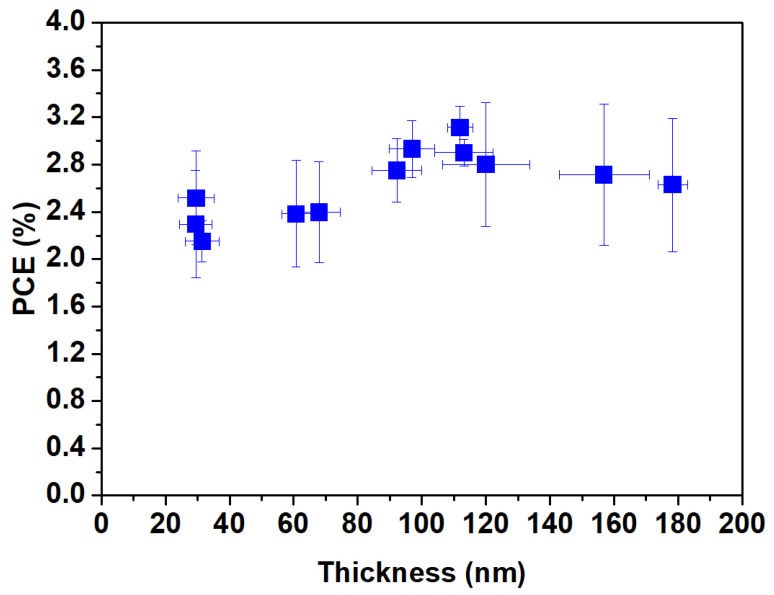
The PCE trend of OPVs fabricated with screen-printed Ag with different film thicknesses of PEDOT:PSS.

In the process of manufacturing OPVs with large-area methods, achieving a larger effective area is essential for mass production. To further increase the area of S-Ag devices, it is necessary to use additional methods for the complete fabrication of the devices. We utilized a slot-die coating process to sequentially complete the coating of the electron transport layer, active layer, and hole-transport layer. We systematically analyzed the suitable thickness of PEDOT:PSS for S-Ag devices. By varying different coating flow rates and speeds, we measured the film thickness values corresponding to different coating parameter combinations and evaluated the efficiency of S-Ag devices to determine the optimal thickness of PEDOT:PSS. The substrate temperature during coating was maintained at 40 °C. Figure 4 shows the efficiency distribution of S-Ag OPV devices with different PEDOT:PSS thicknesses. The results indicate that the thickness of PEDOT:PSS falls in the range of approximately 90–120 nm, and the device efficiency of S-Ag ranges between 2.8 and 3.1%. A PEDOT:PSS film layer that is either too thin or too thick will lead to significant variations in the efficiency of S-Ag devices.

**Figure 5:**
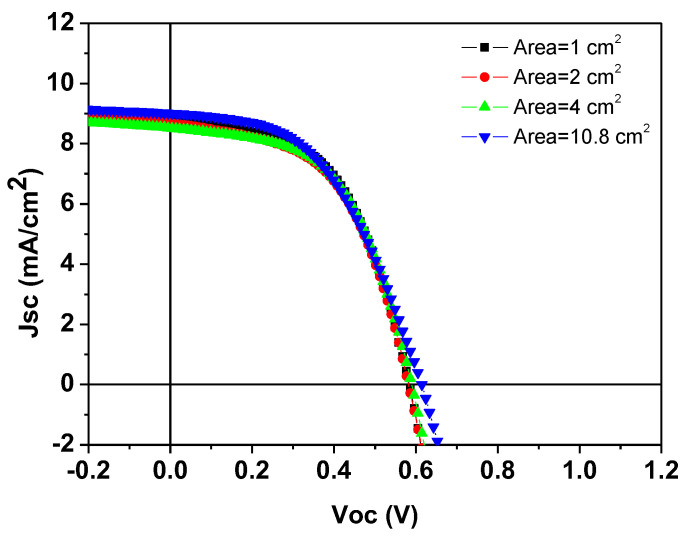
J–V curves of devices with silver electrodes fabricated using the screen-printing process for different areas.

**Figure 6:**
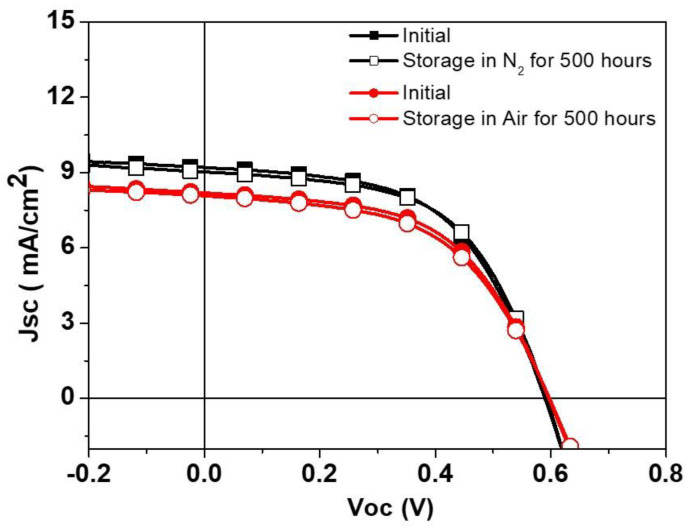
J–V curves of devices fabricated using the screen-printing process, in initial state and after 500 h of dark storage, placed in N_2_ and ambient air.

Through the optimization of the HTL, and adjustment of the silver paste curing parameters, we have successfully fabricated small-area (1 cm^2^) S-Ag devices using the screen-printing process. The PCE of these devices is comparable to that of T-Ag devices. To further verify the PCE changes in S-Ag devices when scaled up, we fabricated large-area S-Ag devices using the screen-printing process, with device areas of 1, 2, 4, and 10.8 cm^2^. The corresponding J-V curves and electrical characteristics are shown in Figure 5 and Table 4. The results indicate that as the device area increased from 1 cm^2^ to 10.8 cm^2^, the PCE of both T-Ag devices prepared by thermal evaporation and S-Ag devices prepared by screen-printing did not significantly decline. This demonstrates the superior uniformity of the PEDOT:PSS film layer prepared by the S2S slot-die coating process and shows that the large-area silver electrodes fabricated by screen printing maintain the same PCE as those prepared by thermal evaporation, even when the device area is scaled up. Additionally, we fabricated modules by connecting four 11 cm^2^ devices in series to evaluate the PCE of modules with different top-electrode fabrication processes. As shown in Table 5, among the five individual modules, the average module PCE of the silver electrodes prepared by thermal evaporation was 2.74%, while that of the silver electrodes prepared by screen printing was 2.62%, indicating a negligible difference between the two modules. Additionally, we monitored the durability of devices fabricated using the screen-printing process. As shown in Figure 6, whether the unencapsulated S-Ag devices were placed in a N_2_ or air environment, their J-V curves showed negligible differences between the initial state and after 500 h. This demonstrates that devices fabricated using the screen-printing process can effectively block moisture and oxygen from the environment, thereby exhibiting excellent stability, which is highly advantageous for practical applications.

**Figure 7:**
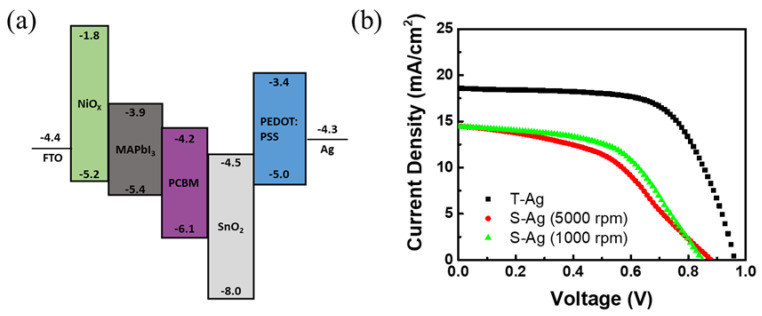
(**a**) The energy–level diagram of the materials used in this PSC device, and (**b**) J–V curves of perovskite solar-cell devices with silver electrodes fabricated using different methods.

Based on the above, we extended the method of fabricating top electrodes on OPVs using the screen-printing process to PSCs. We also compared the effects of different metal-electrode fabrication methods on the efficiency of PSCs. The device architecture of the PSC is FTO\NiO_x_\perovskite\PCBM\SnO_2_ NPs\PEDOT:PSS\Ag, where the fabrication parameters for NiO_x_ and SnO_2_ have been optimized. Similarly, when using the screen-printing process to create electrodes, a good blocking layer is required to prevent the adverse effects of solvents in the silver ink. We referred to the method used in OPVs, utilizing PEDOT:PSS as the blocking layer. The PSCs with silver electrodes fabricated by thermal evaporation achieved an efficiency of 11.23%, which was used as a reference to verify the performance of perovskite solar cells with silver electrodes fabricated using the screen-printing process. However, the PCE value is significantly lower compared to PSC devices without the PEDOT:PSS layer [33], which is clearly due to the energy level misalignment of PEDOT:PSS, as shown in Figure 7a. When fabricating devices with screen-printed silver electrodes, the same process parameters for PEDOT:PSS as those used for thermal-evaporated silver electrodes were initially employed, resulting in a device efficiency of approximately 5.81%. However, based on the PEDOT:PSS parameters developed for OPVs, it was evident that the thickness of the blocking layer needed to be adjusted when using the screen-printing process for silver electrodes. Therefore, the parameter for a thicker PEDOT:PSS layer (~100–120 nm at 1000 rpm) was adopted as the blocking layer. With this adjustment, the efficiency of the S-Ag devices was further improved to 6.64%. The results are shown in Figure 7b and Table 6. The efficiency of the PSCs with S-Ag is still lower than that with T-Ag. In fact, when fabricating silver electrodes for PSC devices using the screen-printing process, we baked the silver electrodes in ambient air. We speculate that prolonged heating time of the perovskite layer in ambient air is the primary reason why the efficiency of the S-Ag devices is significantly lower than that of the T-Ag devices. However, this result demonstrates that material selection and parameter adjustments using the same approach can achieve the goal of fabricating silver electrodes using the screen-printing process. Therefore, developing and optimizing the parameters for the blocking layer can further enhance the efficiency of perovskite solar cells fabricated using an all-solution process.

## 4. Conclusions

In this study, we developed silver electrodes for solar cells using the screen-printing process, thereby achieving the goal of developing solar cells via an all-solution coating process. The experimental results indicate that the selection of blocking layer materials and the optimization of parameters are crucial for the performance of S-Ag devices. In the research on OPVs, by adjusting the thickness of the PEDOT:PSS layer, we were able to achieve efficiency values for S-Ag devices comparable to those of T-Ag devices. Additionally, we employed NIR heating to cure the silver electrodes made by screen printing, significantly reducing the curing time from 30 min required by hot air annealing to just 5 s. Through slot-die coating, we fabricated the electron transport layer, active layer, and hole-transport layer. Even when the device area was increased from 1 cm^2^ to 10.8 cm^2^, the efficiency of S-Ag devices remained comparable to that of T-Ag devices and demonstrated good stability in ambient air. This method was also extended to perovskite solar cells, yielding preliminary results. We believe that with further optimization of the blocking layer materials and process parameters, it will be possible to achieve high-efficiency PSCs with screen-printed silver electrodes.

## Figures and Tables

**Table 1:** Photovoltaic characteristics of OPV devices with thermally evaporated Ag (T-Ag) and screen-printed Ag (S-Ag) electrodes using PEDOT:PSS as the HTL, and with the data averaged over four devices. (The data presented in the table represent the mean values ± standard deviation.)

Device	Jsc (mA/cm^2^)	Voc (V)	FF (%)	PCE (%)
T-Ag	7.70 ± 0.11	0.6 ± 0.01	58.68 ± 1.0	2.70 ± 0.08
S-Ag	6.10 ± 0.43	0.61 ± 0.01	47.57 ± 3.3	1.77 ± 0.23

**Table 2:** Electrical characteristics of OPV devices with S-Ag electrodes under different NIR irradiation intensities, and with the data averaged over four devices. (The data presented in the table represent the mean values ± standard deviation.)

NIR Power (%)	J_sc_ (mA/cm^2^)	V_oc_ (V)	FF (%)	PCE (%)
Ref *	8.84 ± 0.11	0.60 ± 0.00	58.30 ± 0.46	3.08 ± 0.05
**10**	1.79 ± 0.58	0.36 ± 0.31	22.60 ± 4.53	0.15 ± 0.07
**20**	4.29 ± 1.13	0.52 ± 0.10	27.03 ± 1.37	0.60 ± 0.27
**30**	6.99 ± 1.46	0.41 ± 0.16	30.07 ± 7.76	1.00 ± 0.87
**40**	9.34 ± 0.39	0.59 ± 0.00	53.03 ± 1.14	2.90 ± 0.20
**50**	9.40 ± 0.07	0.58 ± 0.02	54.05 ± 3.18	2.90 ± 0.28
**60**	9.57 ± 0.15	0.59 ± 0.01	52.17 ± 1.31	2.97 ± 0.06
**70**	9.25 ± 0.08	0.60 ± 0.00	52.00 ± 0.27	2.87 ± 0.06
**80**	8.45 ± 0.50	0.59 ± 0.01	49.40 ± 0.92	2.50 ± 0.27
**90**	7.40 ± 0.45	0.59 ± 0.04	43.27 ± 1.88	1.87 ± 0.32
**100**	5.32 ± 0.01	0.60 ± 0.01	38.35 ± 1.20	1.25 ± 0.07

* Ref. device: with thermal evaporated Ag as the top electrode.

**Table 3:** Variation of average sheet resistance of silver electrodes with NIR heating.

NIR power (%)	ref	10	20	30	40	50	60	70	80	90	100
Sheet resistance (Ω)	0.39 ±0.02	70.8 ±9.8	65.4 ±6.9	6.2 ±0.92	0.3 ±0.01	0.29 ±0.02	0.22 ±0.01	0.13 ±0.01	0.12 ±0.01	0.12 ±0.00	0.09 ±0.00

ref: refers to the average sheet resistance of silver electrodes obtained after using an oven at 80 °C for 30 min.

**Table 4:** Electrical characteristics of devices with thermal-evaporated and screen-printed silver electrodes for different active areas, and with the data averaged over four devices. (The data presented in the table represent the mean values ± standard deviation.)

Area (cm^2^)	T-Ag				S-Ag			
	J_sc_ (mA/cm^2^)	V_oc_ (V)	FF (%)	PCE (%)	J_sc_ (mA/cm^2^)	V_oc_ (V)	FF (%)	PCE (%)
**1**	9.47 ±0.29	0.61 ±0.01	48.30 ±1.25	2.80 ±0.14	8.79 ±0.34	0.58 ±0.01	54.10 ±2.31	2.80 ±0.14
**2**	8.51 ±0.34	0.59 ±0.01	53.70 ±2.10	2.70 ±0.23	8.70 ±0.49	0.58 ±0.21	53.30 ±3.05	2.70 ±0.24
**4**	8.38 ±0.41	0.59 ±0.01	54.10 ±3.50	2.70 ±0.38	8.52 ±0.42	0.59 ±0.01	54.60 ±3.48	2.70 ±0.34
**10.8**	9.77 ±0.66	0.62 ±0.01	45.00 ±4.60	2.70 ±0.41	9.02 ±0.77	0.61 ±0.01	49.20 ±4.85	2.70 ±0.32

**Table 5:** Electrical characteristics of modules with thermal-evaporated and screen-printed silver electrodes, with individual values for four independent modules.

T-Ag (1 × 1 cm^2^) × 4				S-Ag (1 × 1 cm^2^) × 4			
J_sc_ (mA/cm^2^)	V_oc_ (V)	FF (%)	PCE (%)	J_sc_ (mA/cm^2^)	V_oc_ (V)	FF (%)	PCE (%)
**2.03**	2.39	53.00	2.60	2.28	2.37	48.70	2.60
**2.09**	2.37	59.20	2.90	2.14	2.33	52.70	2.60
**2.01**	2.40	56.20	2.70	2.27	2.38	51.40	2.8
**2.16**	2.39	59.50	3.10	2.06	2.38	53.10	2.60

**Table 6:** Electrical characteristics of perovskite solar-cell devices with silver electrodes fabricated using different methods and with the data averaged over four devices. (The data presented in the table represent the mean values ± standard deviation.).

Device (Top Electrode)	J_sc_ (mA/cm^2^)	V_oc_ (V)	FF (%)	PCE (%)
T–Ag	18.32 ± 0.71	0.96 ± 0.02	63.71 ± 1.46	11.23 ± 1.45
S–Ag/PEDOT:PSS (5000 rpm)	14.09 ± 2.58	0.85 ± 0.03	46.63 ± 4.53	5.81 ± 0.87
S–Ag/PEDOT:PSS (1000 rpm)	14.29 ± 1.13	0.85 ± 0.10	52.41 ± 2.37	6.64 ± 0.79

## Data Availability

Data are contained within the article.
